# Drug Repurposing for Cancers With Limited Survival: Protocol for a Retrospective Cohort Study

**DOI:** 10.2196/48925

**Published:** 2023-11-14

**Authors:** Alejandro Rodríguez-Molinero, Carlos Pérez-López, Jose L Salazar González, Esther Garcia-Lerma, Juan A Álvarez-García, Luis M Soria Morillo, Tomás Salas Fernández

**Affiliations:** 1 Àrea de Recerca Consorci Sanitari de l'Alt Penedès i Garraf Vilafranca del Penedès Spain; 2 Dpto de Lenguajes y Sistemas Informáticos Universidad de Sevilla Sevilla Spain; 3 Biostatistics Unit Bellvitge Biomedical Research Institute (IDIBELL) L'Hospitalet de Llobregat Spain; 4 Agency for Health Quality and Assessment of Catalonia Barcelona Spain

**Keywords:** cancer, death, drug repurposing, epidemiology, mortality, oncology, pharmacoepidemiology, pharmacology, side effects, survival, survivors

## Abstract

**Background:**

Only 5% of the molecules tested in oncology phase 1 trials reach the market after an average of 7.5 years of waiting and at a cost of tens of millions of dollars. To reduce the cost and shorten the time of discovery of new treatments, “drug repurposing” (research with molecules already approved for another indication) and the use of secondary data (not collected for the purpose of research) have been proposed. Due to advances in informatics in clinical care, secondary data can, in some cases, be of equal quality to primary data generated through prospective studies.

**Objective:**

The objective of this study is to identify drugs currently marketed for other indications that may have an effect on the prognosis of patients with cancer.

**Methods:**

We plan to monitor a cohort of patients with high-lethality cancers treated in the public health system of Catalonia between 2006 and 2012, retrospectively, for survival for 5 years after diagnosis or until death. A control cohort, comprising people without cancer, will also be retrospectively monitored for 5 years. The following study variables will be extracted from different population databases: type of cancer (patients with cancer cohort), date and cause of death, pharmacological treatment, sex, age, and place of residence. During the first stage of statistical analysis of the patients with cancer cohort, the drugs consumed by the long-term survivors (alive at 5 years) will be compared with those consumed by nonsurvivors. In the second stage, the survival associated with the consumption of each relevant drug will be analyzed. For the analyses, groups will be matched for potentially confounding variables, and multivariate analyses will be performed to adjust for residual confounding variables if necessary. The control cohort will be used to verify whether the associations found are exclusive to patients with cancer or whether they also occur in patients without cancer.

**Results:**

We anticipate discovering multiple significant associations between commonly used drugs and the survival outcomes of patients with cancer. We expect to publish the initial results in the first half of 2024.

**Conclusions:**

This retrospective study may identify several commonly used drugs as candidates for repurposing in the treatment of various cancers. All analyses are considered exploratory; therefore, the results will have to be confirmed in subsequent clinical trials. However, the results of this study may accelerate drug discovery in oncology.

**International Registered Report Identifier (IRRID):**

DERR1-10.2196/48925

## Introduction

The usefulness of new cancer treatments is traditionally analyzed in clinical trials, which are considered the most rigorous design to study the effects of a treatment on a disease. Despite their strengths, clinical trials have significant limitations, such as cost and time. In a typical clinical trial, one or very few treatments are tested over a course of 7.5 years, and in oncology, the costs range from US $4.5 million (phase I) to US $22.1 million (phase III) [1-4]. Despite this, the clinical trials database ClinicalTrials.gov registers more than 22,000 trials each year, accounting for billions spent annually on clinical trials [5].

In oncology, the results of this substantial financial investment are modest, at best. As an example, only 5% of the molecules tested in phase 1 trials are marketed, and even if they are, they rarely generate a substantial change in the prognosis of patients [6,7]. In addition, the issue seems to be getting worse because the number of approved molecules has not increased in recent decades, but investment by the pharmaceutical industry has increased significantly [8].

Various strategies have been used to try to improve the efficiency of clinical trials. One of the most interesting is drug reuse (drug repurposing), with the aim of finding new uses for existing drugs approved for another therapeutic indication. This approach can, in most cases, allow research to begin with phase 2 trials because preclinical, pharmacokinetic, pharmacodynamic, and toxicity data are usually already known [7]. Looking for new treatments among already known molecules does not seem unreasonable, because in Spain alone, there are more than 2600 active ingredients approved and in use; it makes sense to think that some of them may have unknown effects on cancer. This approach, however, faces significant challenges since the complex and heterogeneous nature of cancer means that drugs with an effect on one type of cancer may not exhibit similar efficacy in several subtypes of cancer. Additionally, issues related to intellectual property, regulatory pathways, and the need for robust clinical evidence contribute to the complexity of drug repurposing efforts in oncology [9,10].

Another approach that substantially lowers the cost of pharmacological discoveries is the use of secondary data for research. Secondary data, which are used in some epidemiological designs, are those that are not collected for the purpose of the investigation but for another purpose, generally before the start of the study. Traditionally, data of this type are of poor quality because they are collected for other purposes, without the rigor of prospective studies. The number of variables may be incomplete because the data collection was not designed to address the research question. However, the technological revolution in recent decades has led to a paradigm shift, leading to reconsiderations of the classic axiom that secondary data are of low quality. New technologies and social networks produce a substantial amount of population data, which are not always of poor quality. The application of these technologies to medicine has led to the implementation of electronic health records and electronic prescription. Although the data in these 2 registries are not research data, they are of high quality, as they are used in clinical practice to manage people’s health. Electronic prescription in Catalonia, for example, is how treatments are prescribed to users of the National Health System; therefore, it contains the drugs, doses, and guidelines actually prescribed to millions of people by their doctors. In addition, these doctors do not type the active ingredients in the prescription, which could lead to errors, but rather select them from lists in drop-down windows; therefore, the database is completely encrypted and does not contain transcription errors, in addition to being continuously updated. Other current databases, such as the mortality registry of the National Institute of Statistics (INE) or the Minimum Basic Data Set (CMBD) registry, where the diagnoses and procedures performed on hospitalized patients in Spain are recorded, can offer sufficient quality, in combination with electronic prescription, to draw conclusions about some of the effects of commercialized drugs [11,12].

Thus, combining the 2 strategies discussed above, we have designed a drug epidemiological study using population databases to identify drugs whose consumption is associated with changes in the survival of patients with cancer. For this, the pharmacological agents consumed by long-term survivors of incurable cancer will be compared with the drugs consumed by patients with shorter survival. We hope that this will allow, with a minimum investment of time and money, the identification of hitherto unknown effects of the molecules marketed in Spain on various oncological diseases.

This paper presents the general protocol for the study; the primary objective is to identify drugs whose consumption influences the survival of patients with high-lethality cancer (defined as those with a 5-year average survival of less than 20%, according to the statistics available at the time of database creation), and the secondary objective is to study the relationship between nonneoplastic diseases of patients with cancer and their survival. Given the diversity of the expected results with respect to different drugs and cancer diseases, we plan to publish the most relevant results in independent papers, in which the methodological details of each substudy will be described, as well as the specific interpretation of the results and their implications, depending on the nature of the findings and the associated cancer.

## Methods

### Design

This will be a retrospective cohort study in which a cohort of patients with high-lethality cancer (cohort 1) and another control cohort composed of people without cancer (cohort 2) will be studied.

### Participants and Settings

The study population will be patients with the following cancers whose estimated survival at 5 years is less than 20%: lung, pancreas, esophagus, gastric carcinomas, hepatocarcinoma, and metastatic cancers.

Cohort 1 will include all patients registered in the databases of the public health system in Catalonia (Spain) who were diagnosed with any of the diseases under study between 2006 and 2012 (Textbox 1 contains the list of International Classification of Diseases, Tenth Revision [ICD-10] codes) and who have complete data regarding the study variables (see “Outcome Measurements and Variables” section).

**Diagnostic codes (International Classification of Diseases, Tenth Revision [ICD-10]) for the pathologies under study.**

**Neoplasm lung**

- C34.0, C34.00, C34.01, C34.02, C34.1, C34.10, C34.11, C34.12, C34.2, C34.3, C34.30, C34.31, C34.32, C34.8, C34.80, C34.81, C34.82, C 34.9, C34.90, C34.91, and C34.92

**Neoplasm pancreas**

- C25, C25.0, C25.1, C25.2, C25.3, C25.7, and C25.9

**Neoplasm hepatic**

- C22, C22.0, C22.1, C22.2, C22.3, C22.4, C22.7, C22.8, and C22.9

**Neoplasm gastric**

- C16, C16.0, C16.4, C16.3, C16.1, C16.2, C16.5, C16.6, C16.8, and C16.9

**Neoplasm esophagus**

- C15, C15.3, C15.4, C15.5, C15.8, and C15.9

**Metastasis**

- C78.0, C78.00, C78.01, C78.02, C78.1, C78.2, C78.3, C78.30, C78.39, C78.4, C78.5, C78.6, C78.7, C78.8, C78.80, C78.89, C79.0, C79.00, C79.01, C79.02, C79.1, C79.10, C79.11, C79.19, C79.2, C79.3, C79.31, C79.32, C79.4, C79.40, C79.49, C79.5, C79.51, C79.52, C79.6, C79.60, C79.61, C79.62, C79.7, C79.70, C79.71, C79.72, C79.8, C79.81, C79.82, C79.89, C79.9, and C80.0

In cohort 2, a total of 4 control participants will be included for each patient with cancer included in cohort 1. Control participants will be selected from those registered in the databases of the public health system in Catalonia whose diagnostic codes do not include neoplasm diseases (ICD-10 codes C00 to D49). The 4 control participants will be matched with the cases in cohort 1 by place of residence and date of birth, of which 2 controls will have been born in the same year and the other 2 controls will have been born 10 years earlier, with the aim of generating an older subcohort with, therefore, higher expected mortality.

Participants who, due to their unique health characteristics, including rare diseases or peculiar sociodemographic data such as extreme ages or residing in sparsely populated areas, were at risk of being identified were excluded from the sample.

### Duration

The patients in cohort 1 will have been diagnosed with the cancer under study between 2006 and 2012, both inclusive, and their survival will be retrospectively monitored for 5 years after diagnosis or until death if it occurs earlier. Cohort 2 will include patients without cancer who have had any type of contact with the health care system between 2006 and 2012, and these individuals will be followed up retrospectively for 5 years or until death if it occurs earlier.

### Data Sources

The study will be carried out using secondary data previously collected from various health databases at the population level. The researchers accessed the data set through the PADRIS program (Programa públic d’analítica de dades per a la recerca i la innovació en salut a Catalunya) of AQuAS (L'Agència de Qualitat i Avaluació Sanitàries de Catalunya) [13]. This program is designed to facilitate research by reusing data from the Catalan Public Utilization System (Sistema Sanitari Integral d’Utilització Pública de Catalunya [SISCAT]) with maximum ethical and data protection guarantees. The data will be pseudonymized and subjected to a study of the risk of reidentification of individuals from the data set, thus minimizing this risk before being used by the researchers.

The general characteristics of the original databases from which the study data will be obtained are summarized below:

- Hospital CMBD is a database in which diagnoses and procedures performed on hospitalized patients are recorded throughout the Spanish territory; all public hospitals and the majority of private hospitals (>95% hospital coverage) are included [14].
- Estació Clínica d'Atenció Primària (ECAP) is an electronic medical history program launched in 2001 and used by family doctors, pediatricians, and nurses from the Catalan Institute of Health (Institut Català de la Salut [ICS]) primary care centers when they visit patients; it includes all previous illnesses known to the primary care physician and records of the health care process in the outpatient setting, including assessments by health care personnel, diagnostic tests, and treatment, among others. The PADRIS program has only provided data since 2006, after which data are considered to be of good quality.
- The registry of causes of death is prepared by the INE using data from the Medical Certificate of Death or Statistical Bulletin of Death, Statistical Bulletin of Judicial Death, and Statistical Bulletin of Childbirth. It is prepared following the criteria established by the WHO (World Health Organization) in the International Classification of Diseases (ICD), which includes more than 12,000 diseases.
- Electronic prescription has been implemented in Catalonia progressively since 2010 and is currently deployed in 100% of primary care centers, pharmacies, and public hospitals in Catalonia. Prescribing and dispensing data will be obtained from the Comprehensive Health Information System of the Catalan Health Service (Servei Català de la Salut [CatSalut]), which is a computerized system used by the doctors of the national health system in Catalonia to prescribe financed pharmacological treatments to their patients and the pharmacies to dispense them; it contains information on the date of the prescription, the active ingredient, and dose, among other variables. Unfunded treatments can be prescribed using this tool but are often prescribed by other means (paper prescriptions).

### Outcome Variables and Measurements

The primary end point is survival, calculated from the date of entry in the database until death or until 5 years of follow-up are reached. For patients in cohort 1, the earliest date of cancer diagnosis for which they have been selected to be part of the cohort will be considered the date of entry into the study (inclusion criterion).

As study variables, the following data will be collected from all patients:

- List of diagnosed diseases (data source: CMBD and ICS ECAP diagnostic registry)
- Date of death (data source: INE cause of death registry)
- Cause of death (data source: INE cause of death registry)
- Pharmacological treatment (data source: Catalan electronic prescription), including all prescribed active ingredients, drug withdrawal dates from pharmacies, consumed doses, and prescribed regimen, during the entire follow-up period
- Sex (data source: Sistema Integral d'Informació de Salut)
- Date of birth (data source: Sistema Integral d'Informació de Salut)
- Basic health area (data source: Sistema Integral d'Informació de Salut)

For the patients included in the study with one of the cancers under study (cohort 1), the following variables will also be collected:

- Type of cancer and presence of metastases (data source: CMBD and ICS ECAP diagnostic registry)
- Date of diagnosis for which the patient is included in the cohort (data source: CMBD and ICS ECAP diagnosis registry)
- Surgical treatment of the cancer (data source: CMBD)
- Date of surgical treatment (data source: CMBD)

### Data Curation

The data used in this study will be obtained from different databases, which must be merged for the analysis. None of the databases will be sorted by patient but by other types of entries (drugs, diagnoses, procedures performed, etc); therefore, the same patient could appear multiple times in each of them. Thus, first, a date will be associated with each event, and the information in the databases will be sorted by patient, with each patient as an entry that appears only once.

The coding of diseases and therapeutic procedures has undergone changes during the years of collection in the cohort, transitioning from using International Classification of Diseases, Ninth Revision (ICD-9) coding to ICD-10 coding. Consequently, all the data with ICD-9 coding will have to be converted to ICD-10 using the conversion tables provided by the Government of Catalonia (Generalitat de Catalunya) on their website [15].

In the pharmacological dispensing database, the active principles will be collected through the Anatomical Therapeutic Chemical (ATC) Classification System, and the amount dispensed monthly will be determined using the number of defined daily doses (DDDs) [16]. DDDs change over the years and are not uniform for different active ingredients or for drugs that form different groups as defined by the ATC4 codes. Thus, when necessary, DDDs will be transformed into units of mass for each of the drugs using the tables provided by the WHO with the evolution of DDDs over time [17].

One of the databases managed includes patients with metastases of any origin. For these patients, their previous diagnoses will be traced, and all the codes for both the CMBD and the ECAP will be analyzed to search for cancer diagnoses before metastasis to determine the origin of the cancer.

Finally, a patient rejection process will be applied based on the consistency of dates between the different databases. Following this principle, all those patients who, having been included in the database, do not have electronic prescription records will be rejected. In addition, all patients without registration or enumeration in the death databases will be excluded. Finally, patients with inconsistent dates will be excluded, for example, with postdeath records in the CMBD, ECAP, or electronic prescription.

### Machine Learning

Machine learning techniques can reveal associations between variables, which are difficult to discover through statistical analyses [18]. Given the large volume of active ingredients to be analyzed and the high number of cases included in the database, in this study, as an additional step to the statistical analyses described below, machine learning techniques will be used to identify associations between the consumption of drugs and survival. The associations identified by machine learning techniques will later be confirmed by the classical statistical analyses described below.

Association rule mining (ARM), widely used for medical data analysis, will be used [19-21]. Through this method, association rules (ARs) are constructed that represent possible relationships between different elements within large data sets, identifying the most relevant ones. ARs consist of an antecedent (left hand side), which can be made up of several elements (drugs, age, etc), and a consequent (right hand side), which is made up of a single element, in the case of this study, survival at 5 years. In this study, a total of 2 sets of different ARs will be built using two approaches: (1) based on the amount of drug consumed, and (2) based on the time of drug consumption.

All variables will be previously categorized for use by the ARM technique. The survival variable will be treated dichotomously, depending on whether the patient has survived for at least 5 years. To obtain the set of rules based on the time of consumption of the drugs (active principles [ATC4] and families [ATC5]), the information will be categorically organized by quarters: whether the drug has been consumed during the quarter or not. To construct the set of rules related to the doses of drug consumed, the average DDD, discretized in 3 quantiles, will be used during the survival time of the patient.

Among all the rules generated by this methodology, the relevant ones will be selected. For this, an a priori algorithm [22] will be used to extract element frequencies, as it has been shown to be effective and is widely used in the literature [23].

Of all the rules produced using this methodology, only those that are met in at least 5 patients will be taken into consideration, and the most relevant will be selected using the following set of metrics:

- Support is a measure that indicates the frequency of the combination of the antecedent and consequent in the data set.
- Confidence is a measure that indicates the probability of obtaining a consequent given an antecedent.
- Lift is a measure that indicates how much more the combination of the antecedent and consequent occurs compared with what would be expected if they were statistically independent.
- Conviction is a measure that evaluates the relationship between the antecedent and the consequent of a rule [24]. This measure takes into account the occurrence of both independently. It is defined as the ratio of the expected frequency of incorrect predictions made by the rule to the actual frequency of incorrect predictions made by the rule. As a general rule, those values that are further from 1 usually indicate the most interesting rules.

First, the data are preprocessed to obtain a column for each active substance in each patient and, depending on the desired screening, the amount of the active substance taken in the different quarters. Other relevant data, such as age and sex, are also added.

Subsequently, AR search algorithms are run on the processed data, with a support threshold set to avoid generating nonrelevant rules, for example, less than 5 patients. However, this support can be filtered again later. Obtaining ARs that indicate static relationships between the different attributes, filtering now those that present survival.

Finally, the filtered rules are sorted by the desired metric (eg, lift). This metric was chosen especially for its ability to find active ingredients with a higher-than-expected survival rate. In the example shown in Table 1, active substance 3 in a medium dose leads to 6 times longer survival than expected in these patients.

**Table 1:** Example of the use of the association rule mining method for the analysis of survival associated with different active ingredients and combinations of active ingredients.

Antecedent			Quantity	Consequents		Support	n		Confidence		Lift	Conviction
**Single active ingredient**												
	active-3	Medium		Survival	0.0079		30	0.5000		6.4054		1.8438
	active-5	Low		Survival	0.0081		31	0.4366		5.5934		1.6364
	active-1	Low		Survival	0.0013		5	0.4166		5.3378		1.5804
	active-4	High		Survival	0.0013		5	0.4166		5.3378		1.5804
	active-2	Low		Survival	0.0021		7	0.4000		5.1243		1.5365
**Combination of active ingredients**												
	active-3 and active-2	Medium, low		Survival	0.0021		7	0.8888		11.3873		8.2974
	active-5 and active-1	Low, low		Survival	0.0015		6	0.7500		9.6081		3.6877
	active-4 and active-2	High, low		Survival	0.0013		5	0.7142		9.1505		3.2267

In addition, it is possible to filter by age, gender, or patient group, as well as search for relationships between them and their survival. Similarly, combinations of active ingredients can be sought that lead to increased survival (Table 1).

### Statistical Analysis

To achieve the primary objective, several exploratory analyses will be performed. These will look for an association between the use of the different drugs available in the database (the exposure variable) and patient survival (the outcome variable). The exposure variable will be treated both dichotomously (exposed or not exposed) and quantitatively. The time exposed to the drug, the average monthly dose, the maximum dose, or the cumulative dose per unit of time will be considered. Specific analyses will be carried out on the effect of 2 or more drugs that have been shown to influence survival when they have been consumed, either simultaneously or sequentially, within the time unit being analyzed. The outcome variable is survival during the observation period. Patients who are censored, for whom no death is recorded during the follow-up time, are considered alive at the end of the period.

In addition to the machine-learning techniques described above, the association between exposure and outcome will be investigated using 2 different approaches. The exposure odds for each drug will be calculated, and this exposure will be compared between patients considered alive at 5 years and those who have died using odds ratios (exposure odds ratio). The group of nonsurvivors will be selected with different time criteria in a sensitivity analysis: deceased in the 1st, 2nd, or 3rd year after diagnosis. Second, the survival time of participants exposed and unexposed to each drug available in the database (individual agents, ATC5 codes) or to each family of agents (ATC4 codes) will be compared.

To avoid bias due to a lack of randomization, in both approaches, matching will be carried out between the groups with respect to confounding variables. In addition, if residual confounding is suspected or an imbalance between groups remains after matching, adjustments for age, sex, and diagnostic groups will be performed. A Cox regression model will be constructed to estimate the effect of the evaluated drug. The hazard ratio (HR) and its 95% CI will be reported. Hazard proportionality will be assessed graphically by Schoenfeld residuals.

As a secondary objective, we plan to analyze the relationship between the presence of various nonneoplastic diseases and the survival of patients with cancer. To do this, we will compare the survival of patients with a specific disease to those who do not have it, adjusting multivariate models for age, gender, other conditions that may impact survival, and medications that have previously been shown to modify survival in previous analyses.

All analyses conducted in the cohort of patients with cancer will be replicated in a control patient group matched by age to the patients with cancer (with expected lower mortality) and in another group of older patients, where each control will be 10 years older than their cancer counterpart (with expected higher mortality). The findings that are reproduced in these groups of the control cohort will be discarded, as they are considered the result of uncontrolled biases or general effects on survival, not related to the cancers under study.

To consider a result statistically significant, the P value will be set at P=.05. All analyses are considered exploratory and hence subject to confirmation in new studies; therefore, no correction techniques will be applied to the P values obtained. All statistical analyses will be performed using R statistical software (version 4.1.0 or higher; R Core Team).

### Methods to Address Potential Sources of Bias

This study, given its retrospective nature, is potentially affected by a series of biases, whose prevention or compensation will be attempted through various techniques. The 3 most important biases are discussed below:

- Reverse causality bias: drugs that are prescribed to patients near death to control their symptoms will appear associated with an increased risk of death in the analyses, with the prescription of these drugs not being the cause of death but a consequence of proximity to death. This bias will be alleviated by eliminating the drugs prescribed 3 months before death from the analysis.
- “Immortal patient” bias: the longer a patient survives, the more likely he or she is to be diagnosed with pathologies and to consume drugs. This can give the false appearance that the drugs consumed throughout life are the cause of prolonged survival. To control this effect, the analyses only considered the drugs consumed up to a certain date in the group of patients who survived until that same date. For example, in the group of patients who have survived at least 3 months after the diagnosis of their cancer, only drugs consumed at any time up to month 3 after the cancer diagnosis will be considered. Thus, the consumption of the drug will not be explained by longer survival because the entire group will have survived until the moment when the drugs under study are consumed. Patients who consume the study drugs after the established time cutoff point will be eliminated from the analysis. As a comparison group, only patients who have not consumed the drug at any point in their disease evolution will be taken into account.
- Diagnostic anticipation or “medical surveillance” bias: this bias occurs when patients are included in studies who are in a screening program for cancer because, in such programs, the cancer is diagnosed earlier, which causes the erroneous impression that the individuals live longer. In our database, patients with certain diseases may have undergone frequent diagnostic tests, thus triggering early cancer detection. For example, patients with chronic obstructive pulmonary disease (COPD) may have undergone periodic radiographs, in which lung cancer has been detected early. This may give the impression that COPD, or the drugs used by patients with COPD, are associated with increased survival from lung cancer. To control this bias, patients with a previous pathology that produces greater medical surveillance of the organ where the cancer developed will be eliminated from the analysis.

### Sample Size Considerations

Based on a maximum 5-year survival probability of 20% (P=.2, *Q*=.8) and with a significance level of 5% (95% CI) and a power of 80%, it will be possible to detect a difference of 10% between 2 groups with 294 individuals per group. Table 2 shows an initial estimate of the number of survivors at 5 years available during the study period. According to preliminary data not shown, the database available for the study includes a total of 102,860 patients diagnosed with the cancers under study.

**Table 2:** Incidence and estimated survival for the main pathologies under study.

Cancer	New cases each year, n	Estimated 5-year survival, n (%)	Cumulative survivors throughout the study, n
Lung	4079	738 (18.1)	3691
Metastatic colon	6128	852 (13.9)	4259
Metastatic bladder	2444	122 (5)	611
Metastatic kidney	1094	131 (12)	656
Metastatic melanoma	860	171 (19.9)	856
Pancreas	1168	96 (8.2)	479
Esophagus	616	116 (18.8)	579
Metastatic stomach	1089	57 (5.2)	283
Liver	670	118 (17.6)	590

### Ethical Considerations

Data handling will comply with Spanish Organic Law 03/2018 for data protection, the General Data Protection Regulation 2016/679, and local data protection regulations. Fundació Unio Catalana Hospitals’ ethics committee reviewed the study (CEI 18/37) and waived patient consent due to anonymized retrospective data review.

## Results

The study is currently in progress, with access to various databases. The data have undergone harmonization and curation, and they are now ready for analysis. We have initiated statistical and machine learning analyses. We anticipate discovering multiple significant associations between commonly used drugs and the survival outcomes of patients with cancer (the primary objective). Additionally, we aim to identify relevant associations between the presence of certain chronic diseases and survival outcomes in different cancer types (the secondary objective). There will be specific effects for particular cancers and general effects that impact the survival of a broad range of cancers. The initial publications containing the first relevant results are expected to be released in the first half of 2024.

## Discussion

The aim of this study is to identify drugs for commercial use for pathologies with an effect on the survival of patients with cancer. For the study, diseases that have high lethality (survival rate <20% at 5 years) will be selected because they are a priority in terms of the search for new therapies and because for these diseases, conventional treatment has had little impact on survival, which facilitates the observation of additional survival effects from other drugs. The diseases have also been selected for their high frequency, so that, despite the high lethality, the group of survivors at 5 years will be large enough to allow comparisons with the deceased group with sufficient statistical power.

Despite the good quality of the data used, this study is based on analyses of secondary data and, therefore, has certain limitations that, although hypotheses can be established, make it necessary for these hypotheses to be tested through trials that generate primary data.

First, we do not have all the variables that can act as confounding factors in patient survival. For example, we do not have data on the histology of the various tumors, which can impact survival. Especially important is also the absence of data on hospital treatment for cancer, which does not appear in any of the available databases. Due to this limitation, the study has been designed to investigate “long survivors” (5 or more years) because, for the diseases studied, there are no treatments that have been shown to extend survival to the fifth year. For this reason, it is not probable that hospital treatments act as a confounding factor (because they have no known association with the outcome studied: survival after 5 years).

Second, patient registration in the databases does not coincide exactly with disease onset, resulting in a shortened estimated time to death. This limitation is greater in the CMBD than in the ECAP databases; therefore, one database will be complemented with the other to alleviate this limitation. However, this temporal bias has an established direction: registration in the databases always occurs after the diagnosis. This allows all cases to be classified correctly in the group of patients identified as long survivors because patients who lived 5 years since their inclusion in the database will have lived at least 5 years from disease diagnosis. However, there will be a classification error whereby some long survivors, for whom their data were entered late in the databases used, will fall within the comparison group (classified as “nonlong survivors”). This error will affect at most 20% of the comparison group because that is the maximum survival at 5 years estimated for the diseases under study. Therefore, the specificity of the classification will be at least 80%, and for some cancers, it will reach 95% (such as metastatic bladder cancer or metastatic stomach cancer). This classification error will bias the results toward the null hypothesis, not invalidating the differences ultimately found between groups, although it does detract from the power of the study. Given that the magnitude of the effect sought is very large, we do not believe that the decline in power due to classification bias prevents the observation of these differences.

Third, despite the shortening of the time frame of the data analysis for each patient to control for reverse causality and “immortal patient” biases, there may be a residual effect derived from these biases within the time frame not eliminated from the analysis; however, we expect this effect to be much smaller. In addition to the aforementioned biases, there may be information biases, mainly due to pathologies not registered in the database. We do not expect that these biases will significantly affect the main variables because cancer diagnoses are usually registered in the databases used as soon as they are known, the pharmacological registry includes drugs dispensed in pharmacies (not only prescribed), and registry mortality is very reliable.

Finally, such a broad exploration of variables and results will lead to the discovery of many random results due to biological variability that may not be able to be replicated in future research. The researchers will consider the implementation of adjustments such as the “false discovery rate.” Notably, the study is exploratory, and the results must be confirmed in subsequent studies designed to test each hypothesis derived from this study.

In conclusion, this will be a pharmaco-epidemiological study with great potential to discover new drugs that influence cancer, but that, by design, will serve only as a preliminary step, guiding subsequent research aimed at the repurposing of active molecules for the treatment of cancer.

## Abbreviations

AQuAS: L'Agència de Qualitat i Avaluació Sanitàries de Catalunya
AR: association rule
ARM: association rule mining
ATC: Anatomical Therapeutic Chemical
CatSalut: Servei Català de la Salut
CMBD: Minimum Basic Data Set
COPD: chronic obstructive pulmonary disease
DDD: defined daily dose
ECAP: Estació Clínica d'Atenció Primària
HR: hazard ratio
ICD: International Classification of Diseases
ICD-10: International Classification of Diseases, Tenth Revision
ICD-9: International Classification of Diseases, Ninth Revision
ICS: Institut Català de la Salut
INE: National Institute of Statistics
PADRIS: Programa públic d’analítica de dades per a la recerca i la innovació en salut a Catalunya
SISCAT: Sistema Sanitari Integral d’Utilització Pública de Catalunya
WHO: World Health Organization

## Data Availability

The databases used in this study are managed by the PADRIS program (Programa públic d’analítica de dades per a la recerca i la innovació en salut a Catalunya) of AQuAS (L'Agència de Qualitat i Avaluació Sanitàries de Catalunya). The authors cannot give access to the aforementioned databases. Any researcher interested in using data from the Catalan Public Utilization System (Sistema Sanitari Integral d’Utilització Pública de Catalunya [SISCAT]) must contact directly the PADRIS program [25].
